# Numerical and Metallurgical Analysis of Laser Welded, Sealed Lap Joints of S355J2 and 316L Steels under Different Configurations

**DOI:** 10.3390/ma13245819

**Published:** 2020-12-20

**Authors:** Hubert Danielewski, Andrzej Skrzypczyk, Marek Hebda, Szymon Tofil, Grzegorz Witkowski, Piotr Długosz, Rastislav Nigrovič

**Affiliations:** 1 Faculty of Mechatronics and Mechanical Engineering, Kielce University of Technology, Tysiąclecia Państwa Polskiego 7, 25-314 Kielce, Poland; tmaask@tu.kielce.pl (A.S.); tofil@tu.kielce.pl (S.T.); gwitkowski@tu.kielce.pl (G.W.); 2 Faculty of Materials Engineering and Physics, Cracow University of Technology, Warszawska 24, 31-155 Cracow, Poland; mhebda@pk.edu.pl; 3 Lukasiewicz Network-Cracow Institute of Technology, Zakopiańska 73, 30-418 Cracow, Poland; piotr.dlugosz@kit.lukasiewicz.gov.pl; 4 Faculty of Mechanical Engineering, ŽilinskáUniverzita v ŽilineUniverzitná 1, 010 26 Žilina, Slovakia; rastislav.nigrovic@snop.eu

**Keywords:** laser beam welding, sealed lap joints of dissimilar materials, austenitic and ferritic-pearlitic steels, numerical simulation, microstructure analysis

## Abstract

This paper presents the results of laser welding of dissimilar joints, where low-carbon and stainless steels were welded inthe lap joint configuration. Performed welding of austenitic and ferritic-pearlitic steels included a sealed joint, where only partial penetration of lower material was obtained.The authors presented acomparative study of the joints under different configurations. The welding parameters for the assumed penetration were estimated via anumericalsimulation. Moreover, a stress–strain analysis was performed based on theestablished model. Numerical analysis showed significant differences in joint properties, therefore, further study was conducted. Investigation of the fusion mechanism in the obtained joints wascarried out using electron dispersive spectroscopy (EDS) and metallurgical analysis. The study of the lap joint under different configurations showed considerable dissimilarities in stress–strain distribution and relevant differences in the fusion zone structure. The results showed advantages of using stainless steel as the upper material of a microstructure, and uniform chemical element distribution and stress analysis is considered.

## 1. Introduction

A number of analytical, numerical, and empirical studies have shown the potential of laser beam welding (LBW). First commercially applied in 1970 [1,2,3], LBW has become one of the most widely studied techniques of joining metallic parts.A focused laser beam causes rapid vaporization and ionizationof the metal. The keyhole effectallows lap welding [4,5]. The weld bead geometry and the joint properties are related to the process parameters and the properties of the welded materials [6,7]. Some process parameters depend on the laser type used (wavelength, transverse mode, pulse/continuous-wave operating mode), the laser machine system (spot size, single or multi-spot optics, focal length), and the programmed parameters (emission frequency, output power, welding speed, focal point position). The material properties, such as thermal conductivity, specific heat capacity, latent heat, and thermal diffusivity, affect the welding dynamics andoutcome [8]. The laser beam penetration is related to the surface reflectivity and the ionization effect of the metalvapor, therefore, the intensity of plasma generation varies for different materials [9]. The beam penetration through two materials is a complex phenomenon, moreover, in dissimilar lap joints, the results of the welding process depend on the welded material configuration.

Numerous studies of laser welding technology, including lap welding, are being carried out. Many of those studies focus on joining special application materials such as titanium, aluminum and nickel-based alloys, zinc-coated steels, or a combination of these materials [10,11]. However, as theresearchersfocus on one configuration type, there is a lack of publications showinghow joint properties change under different configurations. Therefore, the authors presented acomparative study of sealed lap joints with partial penetration, where low-carbon and stainless steels are welded alternately using a laser beam [12]. The joints are intended for use in pipeline components and large crude oil storage tanks, where high joint quality and strength are critical characteristics [13,14].

Nowadays, the development of computing power makes it possible to perform advanced calculations of welding processes, based on the finite element method (FEM) and computer-aided design (CAD) geometry, divided into finite elements (FEs). By solving the heat transfer problem, using differential equations, many physical phenomena, including phase transformation and different heat transfer mechanisms, can be taken into account [15,16,17]. Nevertheless, in numerical simulations, some parameters cannot be used in the model directly as input data, and some simplificationsare necessary [18]. Simple numerical models of laser welding provide a thermal solution where the fusion zone (FZ) and the heat-affected zone (HAZ) dimensions can becalculated. Nevertheless, a more advanced thermo-mechanical solution, a stress–strain analysis, can be carried out. The simulation of a lap joint is complex, and the complexity of the process increases when materials with different thermophysical material properties are to be joined. Realistic results can be obtained by developing an accurate heat source (HS) model andadequate boundary conditions [19,20].

The welding of dissimilar materials is problematic, especially when a sealed lap joint is considered. Many publications report the study of joint properties based on process parameters, while neglecting other aspects [21]. In this paper, the authors proposed a new approach to the problem, by analyzing the joint depending on the configuration of welded materials. On the basis of a numerical simulation, welding parameters were estimated, and stress–strain analysis was performed. The structure of the welds wasstudiedusing metallographic and electron dispersive spectroscopy (EDS) analyses. The researchshowedadvantages and disadvantages of austenitic and ferritic-pearlitic steels welded under different configurations ina sealed lap joint.

## 2. Material and Experimental Design

### 2.1. Materials

The materials used in the experiment were 4 mm thick steel sheets in grades S355J2 (according to EN 10025-2 [22]) and 316L (according to ASTM A240 [23]), with dimensions of 50 mm × 20 mm. The S355J2 steel is a commonly used, unalloyed, low-carbon construction steel with a ferritic-pearlitic structure. The other material is austenitic stainless steel 316L, with a high content of chromium and nickel. Both steels are characterized as materials of good weld ability, however, the differences in the chemical composition (specified in Table 1) affect the welding process and the properties of the joints.

**Figure 1:**
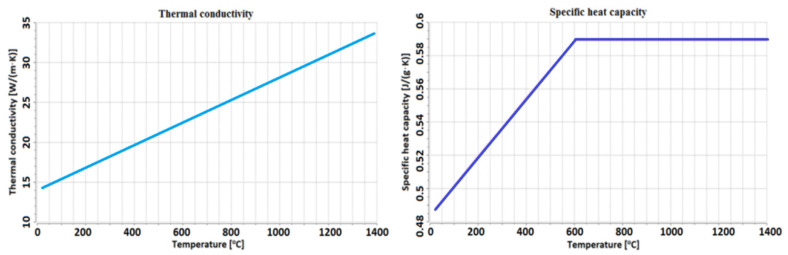
The temperature-dependent thermo-physical properties of 316L steel.

**Figure 2:**
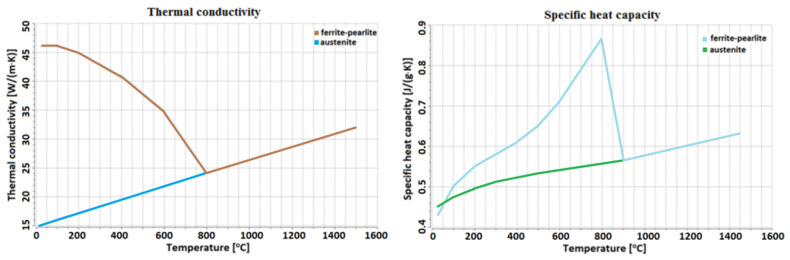
The temperature-dependent thermophysical properties of S355J2 steel.

The materials used have different thermophysical properties that are not constant and change with temperature (Figure 1 and Figure 2). This phenomenon affects the laser beam absorption, which increases with material temperature during welding and plays a significant role in the process dynamics. Figure 1 and Figure 2 show the material database with the modified specific heat used to model the latent heat effect, calculated using JMatPro (Sentesoftware, Guildford, Surrey, UK) included in the Simufact Welding 8 software library (MSC Software Company, Hamburg Germany).

The thermophysical properties for the abovementioned materials have a dissimilar range. For austenitic steel, the thermal conductivity uniformly increases in the range of 14 to 34 W/(m·K). Moreover, the specific heat capacity has linear characteristics in the range of 0.49 to 0.59 J/(g·K), and from 600 °C it is linearhorizontal. However, for low-carbon steel, these properties are related to the material phase, and for the austenitic phase, the linear dependence of thermal conductivity in the range from 15 to 33 W/(m·K) can be observed. On the other hand, for the ferrite and pearlite phase, the thermal conductivity decreases from 46 W/(m·K), until it reaches 800 °C, where it overlaps within austenite conductivity. In S355J2 steel, for the austenitic phase, specific heat capacity increases from 0.3 to 0.62 J/(g·K), with a characteristic similar to linear for the ferrite and pearlite phases, and specific heat capacity rapidly increases to 800 °C, reaching the value of 0.86 J/(g·K), and then rapidly decreases; from 900 °C, it overlaps within the austenite value.

### 2.2. Numerical Simulation Procedure

**Figure 3:**
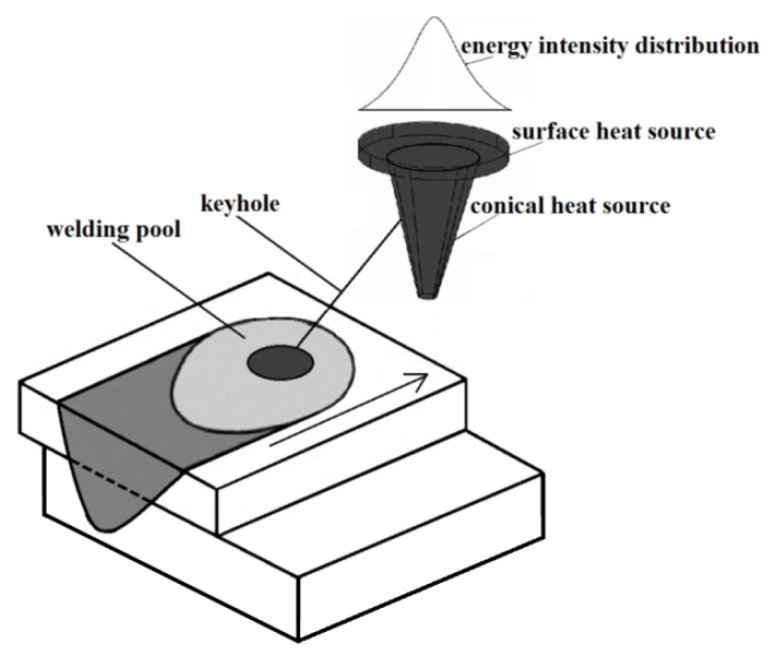
Heatsource model of laser lap joint welding.

For a programming simulation of the welding process, particularly LBW, calibration of the HS model is required and, therefore, at the preliminary stage, by comparing the results of trial welding with the simulation, an accurate model was obtained [24]. Softwarecommonly used for welding simulationsare based on solving heat-mass flow phenomena (ANSYS with the Fluent module (Ansys Inc., Southpointe, Canonsburg, PA, USA), software with an additional welding module (Abaqus), and software dedicated towelding applications, such as SYSWELD and Simufact Welding [25,26]. From the aforementioned software, Simufact Welding was used for estimating welding parameters, and performing stress and strain analysis. The selected program, based on Marc solver (MSC Software Company, Hamburg Germany), is software dedicated to welding applications, and provides sufficient accuracy of results with a relatively quick calculation time. Simulations of conventional arc welding use the double-ellipsoid Goldak model, however, simulations of laser welding are based on volumetric heat sources (conical and cylindrical), with uneven power distribution (Gaussian parameter). Moreover, some methods use a combination of HSs, with a conical (1) source for simulating the keyhole effect, and a disc-shaped source for simulating laser beam absorption by the material surface (Figure 3) [27,28].

Conical volumetric heat source with the Gaussian distribution can be described by the following equation:

(1) $$Q\left(x,y,z\right)=Q_{0}\exp\left(-\frac{x^{2}+y^{2}}{{\left(r_{i}+\frac{r_{t}-r_{i}}{z_{t}-z_{i}}\left(z-z_{i}\right)\right)}^{2}}\right)$$

where $Q_{0}$—the maximum heat flux density in a volumetric heat source, $r_{t}-r_{i}$—the dimensions of the upper and lower conical radius, $z_{t}-z_{i}$—the depth of the conical heat source, *x*, *y*, *z*—the heat source coordinates.

Solving the governing heat equation based on Fourier’s law for three-dimensional heat conduction (2), witha partial differential equation in a nonlinear form, is done with the following equation:

(2) $$\rho c\left(T\right)\frac{\partial T}{\partial t}=\frac{\partial }{\partial x}\left(k\left(T\right)\frac{\partial T}{\partial x}\right)+\frac{\partial }{\partial y}\left(k\left(T\right)\frac{\partial T}{\partial y}\right)+\frac{\partial }{\partial z}\left(k\left(T\right)\frac{\partial T}{\partial z}\right)+q_{v}$$

where *c(T)*—temperature-dependent specific heat capacity; *k(T)—*temperature-dependent thermal conductivity; $q_{v}$*—*volumetric internal energy; *x, y, z—*space coordinates; *T—*temperature; ρ *—*density; and *t—*time.

The conical heat sourcecan be described as follows:

(3) $$q_{l}\left(x,\;y,\;z\right)=\frac{9\eta_{l}P_{l}e^{3}}{\pi \left(e^{3}-1\right)\left(z_{t}-z_{i}\right)\left(r_{t}^{2}+r_{t}r_{i}+r_{i}^{2}\right)}\exp\left(-\frac{3\left[{\left(x-vt\right)}^{2}+y^{2}\right]}{\left(r_{t}-\left(r_{t}-r_{i}\right)\frac{z_{t}-z}{z_{t}-z_{i}}\right)}\right)$$

where $\;q_{l}$—heat flux, $z_{t}-z_{i}\;$z— coordinates (heat source depth), $r_{t}-r_{i}$—upper and lower conical radius, e—natural logarithm, $r_{t}-\left(r_{t}-r_{i}\right)\frac{z_{t}-z}{z_{t}-z_{i}}$—linear decrease in distribution along theconical heat source, $P_{l}$—laser power, $\eta_{l}$—laser heat source efficiency.

Thermal conductivity, specific heat, and emissivity in the heat transfer analysis depend on the temperature, however, the used model is based on the assumption that mass density is constant. By an extrapolating or interpolating procedure, the temperature-dependent properties are averaged. Latent heat is related to phase transformation from solid to liquid metal, or vice versa. Phase change modeling is a complex process, however, using simplification, where latent heat is uniformly released in the solid–liquid range, it can be calculated [29,30].

Due to the convection–diffusion effect involved, the Petro–Galerkin model with nodal velocity vectors was used, which can be described as follows:

(4) $$\frac{\partial T}{\partial t}+v\cdot \nabla T=\nabla \cdot \left(\kappa \nabla T\right)+Q$$

where v—nodal velocity vector, *T*—temperature, κ—diffusion tensor, Q—source term.

The surface energy is determined by calculating the thermal energy that affects the boundary conditions, including thermochemical ablation. These phenomena affect the convective heat flux, and the mass and the enthalpy flow are related to molecular diffusion. The surface energy is correlated with the heat transfer to the material by conduction through a heat-mass flow towards the surface as a result of the evaporation of the material.

**Figure 4:**
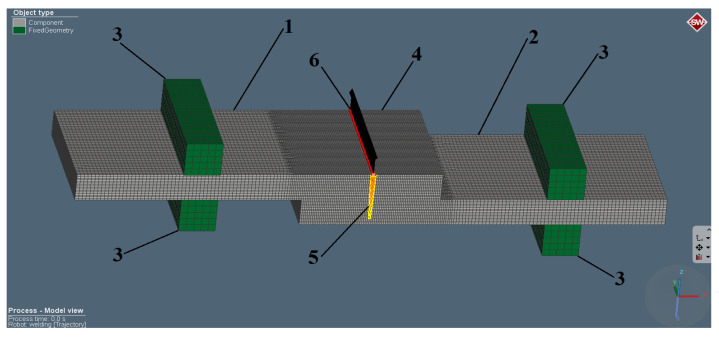
Laser welding finite element (FE) model with defined elements: 1—topsheet 2—bottom sheet, 3—fixed bearings,4—refined FE area, 5—heat sources, 6—welding trajectory.

Specimens with a size of 50 mm × 20 mm were used for the FE model. Three-dimensional solid hexahedral finite elements were used while meshing. The sizes of the elements were determined by adjusting the resolution and accuracy of the temperature distribution in the regions of severe thermal gradients. A mesh convergence study was performed, within FE sizes of 1.0000, 0.7500, 0.5000, 0.2500, 0.1250, and 0.0625 mm. For 0.0625 and 0.1250 mm, no relevant differences in weld geometry and heat distribution were observed, however, there were some differences between 0.1250 and 0.2500 mm. Therefore, the nominal FE size was set as 0.2500 mm, and in an area where a temperature exceeding 400 °C may occur (Figure 4, region 4), an FE refinement was performed, and the FE sizewas set as 0.1250 mm. Two 4 mm thick sheets (Figure 4, elements 1 and 2) were oriented in a lap configuration and fixed in three-dimensional space by additional elements (Figure 4, element 3). The welding trajectory was set in the center of the refinement area (9.5 mm from the sheet edge).

The geometries of the heat sources (Figure 3) (3), are related to the used welding optics. In this case, the focal length was equal to 270 mm with a focal point diameter of 0.3 mm. Nevertheless, the HS geometry calibration for obtaining more accurate results was required. Therefore, a trial calibration weld at the speed of 1 m/min and output power equal to 4 kW was performed (to achieve a keyhole effect). A comparison with the simulation results showed a small discrepancy (width of the face of the weld, with anerror less than 15%), and so the heat source geometry was adjusted until the error was less than 1% [31,32,33]. According to the preliminary calibration performed, a conical heat source with a depth equal to 7 mm, upper radius r_t_ equal to 0.4 mm, and lower radius r_i_ equal to 0.2 mm, as well as a disc-shaped heat source, with a radius equal to 0.5 mm and depth equal to 0.05 mm, were programmed. The volume heat fraction defined the laser power division between the conical and disc HS and for laser keyhole welding, the value was set as 0.95, which means that 95% of the total power was assigned to the conical HS. Moreover, the Gaussian distribution parameter for the disc HS was defined as 1.0 and for the conical HS as 2.8, and they are related to TEM01* (CO_2_ laser, transverse mode). The materials used in the simulation were structural steel ofgrade S355J2 and 316L austenitic stainless steel. Laser welding with a keyhole effect makes it possible to obtain deep material penetration, however, for a CO_2_ laser, the metal surface has high reflectivity and, therefore, HS efficiency for S355J2 was assumed as 0.6, and for 316L as 0.7. The programmed efficiencies are related to laser beam interaction with the materials used, where the reflectivity and the ionization effect have a significant influence on those aspects [34,35,36].

**Figure 5:**
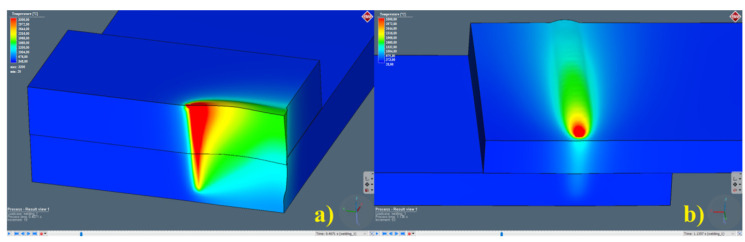
Simulation of laser lap joint welding using the deep penetration mode: (**a**) view of the keyhole effect in the cross-section, (**b**) top view of a moving keyhole, and weld formation.

The simulation of dissimilar, sealed, lap joint laser welding, for two configurations of the top material, was carried out using the Simufact Welding software. Simufact solves a heat transfer problem in solid material, considering the convection–radiation effect, however, no mixing effect of welded material can be taken into account. Figure 5a shows a keyhole in the cross-section during the lap welding, and Figure 5b shows the top hat of the keyhole, during the formation of the face of the weld. The results are presented for the temperature scale in the range of 20 to 3070 °C (from normal condition to the boiling point).

The first joint configuration assumes low-carbon S355J2 steel placed on the top, and the second configuration assumes the opposite: S355J2 steel on the bottom and 316L stainless steel on the top. The research assumed obtaining a sealed lap joint, with partial weld penetration in the bottom sheet [37,38]. Numerical simulations of laser welding were performed at a constant speed of 1 m/min and variable output power in the range of 3 to 6 kW, which increased with each subsequent step by the value of 0.5 kW until the assumed penetration was obtained. The heat source operating time, based on the HS speed and the trajectory length, was equal to 1.2 s, however, the full simulation time, including cooling, was programmed as 30 s. According to the performed simulations, the obtained weld bead geometry and stress–strain distribution (according to the determined measurement points) were studied.

### 2.3. Experimental Welding Procedure

The first step of the experimental research was the HS validation. For this purpose, a preliminary test according to the procedure described in Section 2.2 was carried out. Based on the aforementioned procedure, calibration of the HS geometry and the efficiency coefficient was carried out. The parameters estimated in the numerical simulation, welding speed equal to 1 m/min and output power 6 kW, were used to perform trial welds with a TrumpfTruFlow6000 CO_2_ CW laser (wavelength 10.6 µm) (Trumpf Group, Ditzingen, German), using welding optics with the focal length of 270 mm and the spot diameter equal to 0.3 mm. This type of laser has worse parameters than fiber or disc lasers, however, further planned research requires the use of single and twin spot optics for an extended weld area, which are available for this type of laser. To shield the welding zone, helium (5.0) was used, with the flow rate equal to 20 l/min conveyed coaxially. The laser beam was focused on the surface of the top plate in a PA (flat) position and the welding trajectory was established based on the boundary conditions of the simulation [39]. Using single pass welding, lap joints in two configurations were obtained: the 1st with the low-carbon steel placed on the top (and the 316L sheet at the bottom), and the 2nd with the 316L steel placed on the top (and the S355J2 sheet at the bottom). Welding procedure qualification was performed according to PN-EN ISO 15609-4: 2009 [40] and the joint quality level was specified according to PN-EN ISO 13919-1 [41]. The results of the simulation and the metallographic analysis of the trial joints were studied.

### 2.4. Microstructure Analysis

Validation of the simulation results is related to a weld bead build analysis, therefore, measurements of characteristic geometries were carried out. Specimens for further analysis were prepared by cutting them in half (according to the cross-section of the weld), polishing, and etching with Adler reagent. A metallographic analysis of weld structures according to PN-EN ISO 17639 [42], using a Hirox KH-8700 (Hirox Co Ltd., Tokyo, Japan) confocal digital microscope and a scanning electron microscope JSM-7100F (JEOL Ltd., Tokyo, Japan), was carried out. By visual tests and weld bead build evaluation, welding defect detection and inclusion analysis wasperformed. Investigation of the weld uniformity by element distribution analysis, using a JEOL scanning electron microscope with an electron dispersive X-ray spectroscopy analyzer, was carried out [43].

## 3. Results

### 3.1. Global Observation

**Figure 6:**
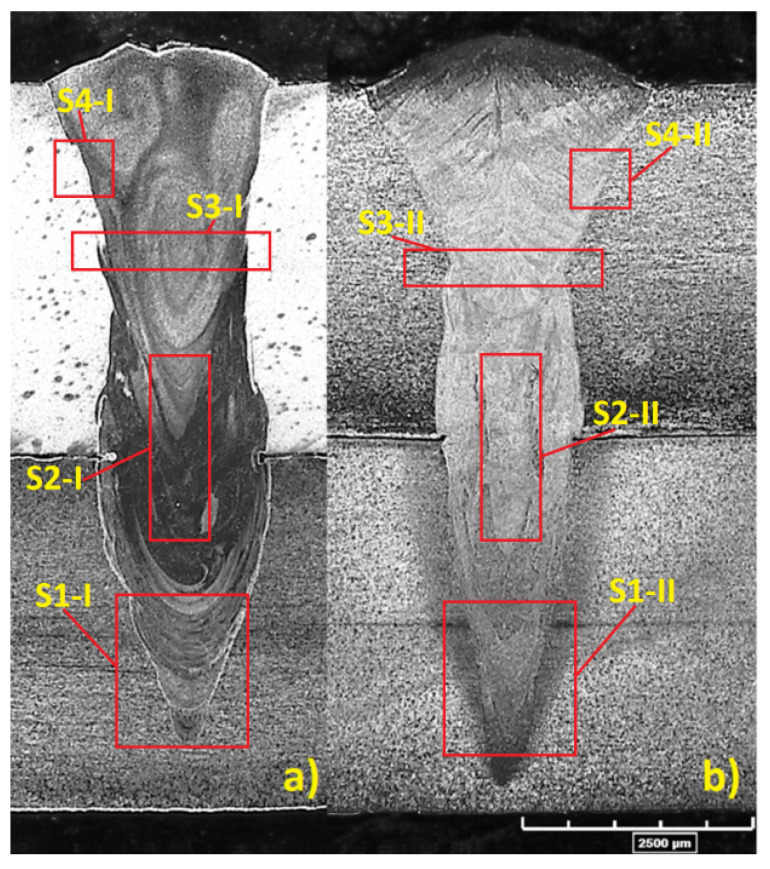
The build of welds with the region defined for further investigation, (**a**) 1st joint configuration (S355J2 steel on the top), (**b**) 2nd joint configuration (316L steel on the top).

Due to the significant mismatch of thermal conductivities, specific heat capacity, and surface absorptivities in the welded materials, the FZ and HAZ are different. Moreover, a numerical simulation analysis showed further differences. The research aimed to obtain sealed lap joints, with a bottom sheet welded approximately to 3/4ths of its thickness. A macroscopic analysis showed only partial material mixing in the bottomsheet (Figure 6). The decision of which configuration to choose for better properties is complex and requires an extended thermalstress–strain numerical analysis, as well as micro- and macro-structure examinations. The preliminary visual tests (VTs) showed a good weld build, lack of defects, a convex face of the weld, and assumed partial penetration of the bottom plate was obtained, therefore, according to therequirement of the PN-EN ISO 13919-1 standard, a B quality level was obtained [44,45].

In the obtained welds beads, some separate lap joint characteristic regions were indicated, where: S1—root of the weld, S2—intersheet area, S3—uniform mixing of top plate weld area, S4—HAZ region (Figure 6). Potentially non-uniform mixing of fused materials was detected, which can affect the joint strength and corrosion resistance and can lead to micro-cracking. Therefore, a microstructure analysis, extended to include a numerical stress–strain study for lap welds evaluations, was carried out [46,47].

### 3.2. Numerical Simulation

The weld bead geometry based on the solid–liquid range was analyzed. On the basis of the programmed HS parameters and established boundary conditions, a numerical simulation of laser lap welding was performed at a constant speed and varying output power. The total thickness of the materials positioned in the lap configuration was equal to 8 mm. As sealed joints were to be obtained, the partial penetration of the weld was up to 7 mm at an output power equal to 6 kW. The differences in the welded materials, especially the absorption coefficient, the thermal conductivity, and the vaporization–ionization effect, made it necessary to change the HS efficiency for each simulation configuration. Therefore, the aforementioned HS efficiency for the low-carbon steel was adopted as 0.6 and for the stainless steel as 0.7 [48,49].

**Figure 7:**
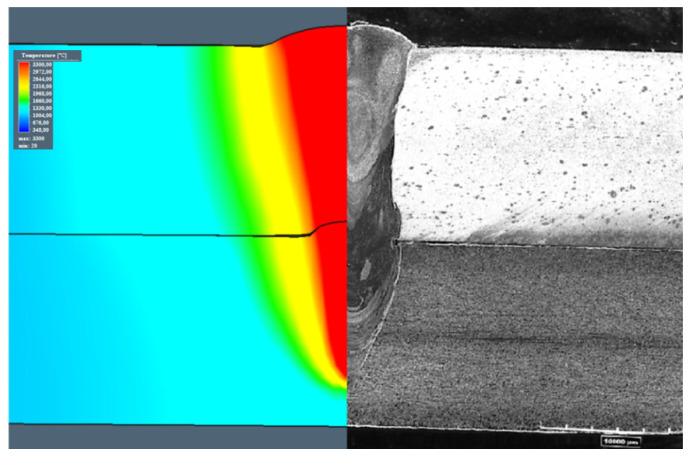
Comparison of the simulation and the trial joint weld build—1st configuration (S355J2 steel on the top).

**Figure 8:**
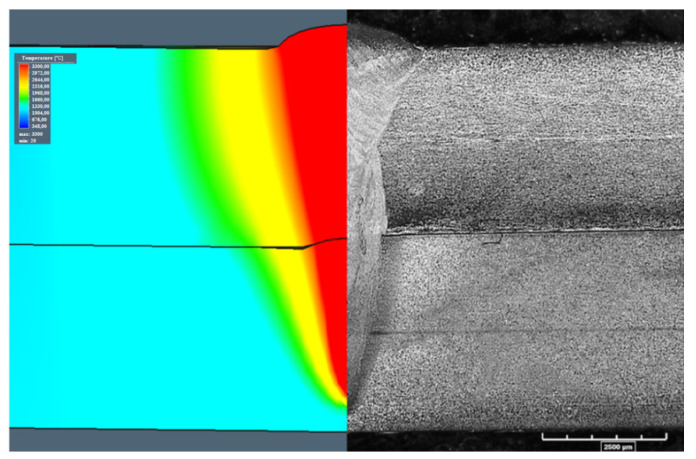
Comparison of the simulation and the trial joint weld build—2nd configuration (316L steel on the top).

For the 1st configuration, aweld depth equal to 7.1 mm, a weld face width equal to 2.87 mm, and a width of the overlap region equal to 1.92 mm were calculated, with experimental values equal to 7.16 mm, 2.51 mm, and 1.83 mm, respectively (Figure 7). The calculation results for configuration 2 showed similar values, where the depth was equal to 7.15 mm, the weld face width to 3.1 mm, and the width of the overlap region to 1.7 mm. The experimental values were equal to 7.3 mm, 3.15 mm, and 1.59 mm, respectively (Figure 8). Some differences in the weld build were observed. The weld obtained in the 2nd configuration had a typical U-groove weld build, with a wider face of the weld, however, the weld waist was narrower. The 1st configuration showed a narrower face of the weldand a wider weld waist.

Figure 7 and Figure 8 compare the simulation and experimental results. The predicted weld bead geometries were compared to the measurements obtained from the trial joints [50]. The results showed good agreement between the predicted and measured characteristic dimensions of weld geometries.

The studied weld bead build showed some differences but no welding defects or incorrect weld structures were observed. Therefore, in order to decide which configuration gives better results, a further study, starting from a stress–strain distribution analysis, was carried out.

**Figure 9:**
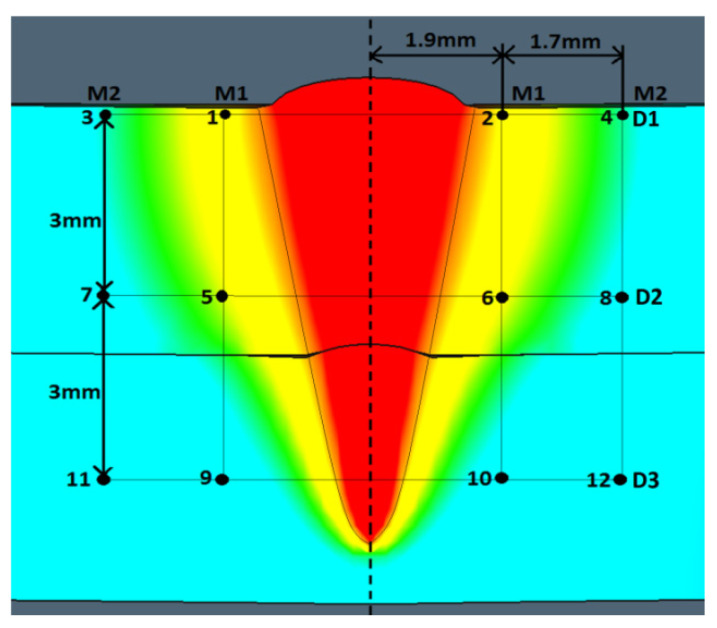
Defined points and lines for stress–strain numerical analysis, selected in critical joint areas.

Considering the stress and strain analysis, it is relevant to study the variability of those phenomena in time. Therefore, measurement points, located across determined lines (characteristic joints areas), as shown in Figure 9, were used to perform the analysis, referred to the welding and cooling stages [51,52,53].

**Figure 10:**
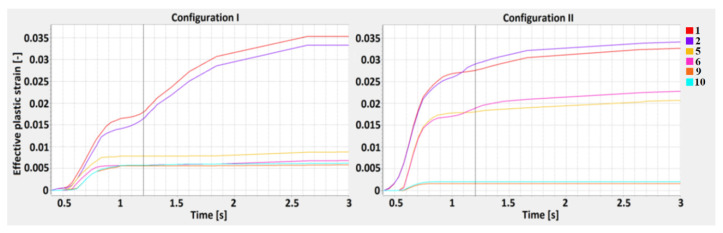
The effective plastic strain curve for the 1st (S355J2 steel on the top) and 2nd (316L steel on the top) configurations along the M1 line.

**Figure 11:**
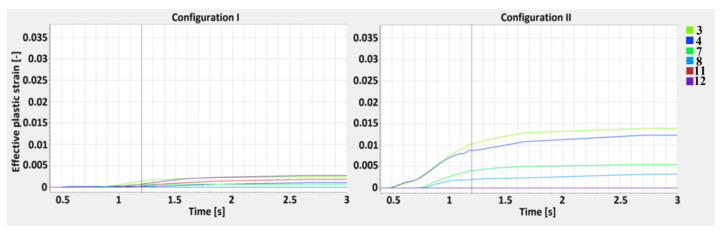
The effective plastic strain curve for the 1st (S355J2 steel on the top) and 2nd (316L steel on the top) configurations along the M2 line.

**Figure 12:**
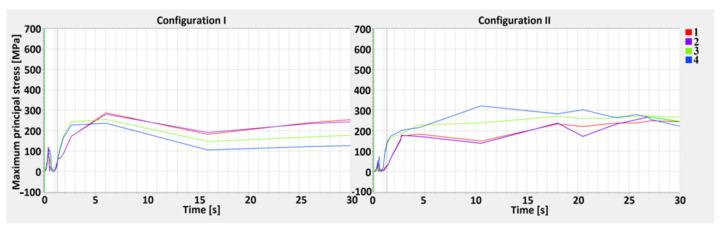
The maximum principal stress curve for the 1st (S355J2 steel on the top) and 2nd (316L steel on the top) configuration along the D1 line—top.

**Figure 13:**
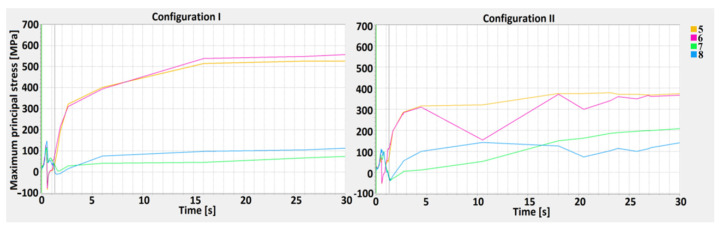
The maximum principal stress curve for the 1st (S355J2 steel on the top) and 2nd (316L steel on the top) configuration along the D2 line—central.

**Figure 14:**
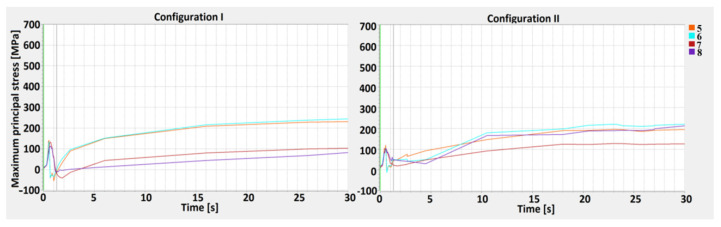
The maximum principal stress curve for the 1st (S355J2 steel on the top) and 2nd (316L steel on the top) configuration along the D3 line—bottom.

The measurement points (1–12) were defined along the M1 and M2 measurement lines—parallel to the weld axis, where M1 is the inside line (near fusion line) and M2 is the outside line (near the HAZ line), and D1, D2, and D3 lines are perpendicular to the weld axis, where D1 is the top line, D2 is the central line, and D3 is the bottom line of weld profile. Based on the defined measurement points and lines, a stress–strain analysis for the 1st and 2nd configurations was carried out (Figure 10, Figure 11, Figure 12, Figure 13 and Figure 14).

Effective plastic strain is a monotonically increasing scalar value which is calculated incrementally, as a function of the plastic component of the rate of the deformation tensor. The effective plastic strain increases whenever the material is actively yielding [54]. An analysis of the effective plastic strain results is shown below, according to the defined M-lines. Welding time alone (1.2 s) during the performed simulation is shown by the perpendicular line.

The tensorial strain values are not monotonically increasing as they reflect the current, the total (elastic+plastic) state of deformation, primarily in the HAZ and across the fusion line where, due to the constituent thermal expansion mismatch, the phase transformation is the most intense. The value of effective plastic strain is the integral of stepwise increments of plastic deformation continuing for a period of time; therefore, the analyzed values are shown according to the welding time. The results show the highest effective plastic strain value across the M1 line and are similar for both joint configurations, however, in the 2nd case, the curve is sharper. The values obtained across the M2 line show greater differences and, in the 2nd configuration, they were almost 4.7 times greater than in the 1st configuration. While the values for the M1 line obtained at the surface are similar (points 1 and 2), in the central zone of the 1st configuration, they are equal to the bottom zone values (points 5 and 6). For the 2nd configuration, three separate regions of effective plastic strain can be identified. The computation times of the welding process (without cooling) were equal to 1.2 s, therefore, for these specific points, some changes in the plots can be observed [55,56,57].

A maximum principal stress (MPS) analysis, concerning values changing in time, was performed as well (Figure 12, Figure 13 and Figure 14). The points selected for the MPS analysis were related to the weld depth (D-lines). By performing a distribution analysis of the maximum principal stress, the areas of stress concentrations can be identified. These particular areas show where crack propagation could start when critical material parameters are reached. An analysis of the results can shown whether in particular joints configurations, the MPS did not exceed the critical values, disqualifying one of the lap joints [58,59].

Analysis of MPS showed higher stress concentrations across the D2 line, particularly in points 5 and 6 (in the middle of the HAZ). The greatest calculated values, equal to 540 MPa, occurred in the 1st configuration, where low-carbon steel is located on the top. In the 2nd configuration, the highest stress occurred across the D2 line as well, however, it did not exceed 400 MPa. At the surface (D1 line), the maximum principal stress exceeded 300 MPa only in point 4. Across the D3 line, the maximum principal stress is similar in both configurations, with slight differences in austenitic steel (in 1st configuration). An increase in the maximum principal stress in D1 and D2 occurred after the welding, and MPS values were related to the movement of the HS, and increased rapidly during the cooling stage, after the heat source was turned off [60,61,62,63]. One must keep in mind that the measurement points were located on the sectional plane in the middle of the welding trajectory.

### 3.3. Metallographic Analysis

Important differences between stress–strain distribution showed significant dissimilarity in joints properties, however, considering only simulation, it is hard to define which configuration provides better results, and further analysis is required. Therefore, the microstructure of characteristic weld areas, chosen alloyed elements distribution (Figure 21), and the precipitates in the fusion zone were studied and the results are shown below.

**Figure 15:**
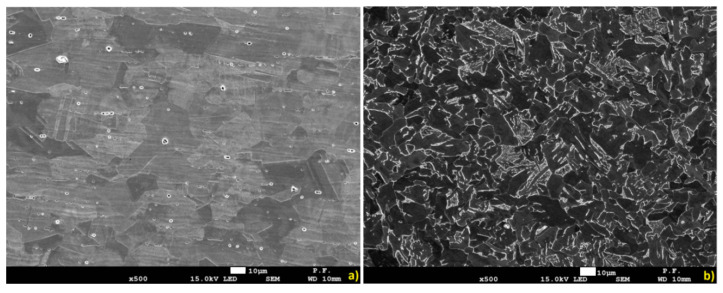
The microstructure of welded base materials: (**a**) 316L steel, (**b**) S355J2 steel.

**Figure 16:**
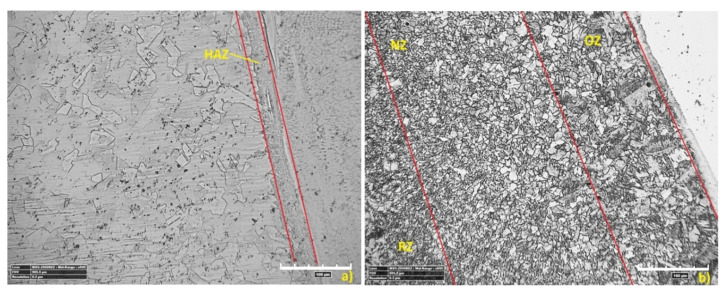
The microstructure of the heat-affected zone (HAZ): (**a**) 316L steel, (**b**) S355J2 steel.

Figure 15 shows the microstructure of the base material (BM). The structures shown are typical for the used materials, with a ferritic-pearlitic microstructure of low-carbon S355J2 steel (Figure 15a) and an austenitic structure of stainless 316L steel (Figure 15b). The HAZ areas (Figure 16) in both materials are different. In 316L steel, the HAZ is narrow, with grain refining along the fusion line, and an increase in the volume fraction of ferrite δ. The HAZ identified in S355J2 steel is much wider and is made up of three separate regions: overheated zone (OZ), normalization zone (NZ), and partial recrystallization zone (RZ) [64,65]. The HAZ was investigated according to the S3 region (Figure 6).

**Figure 17:**
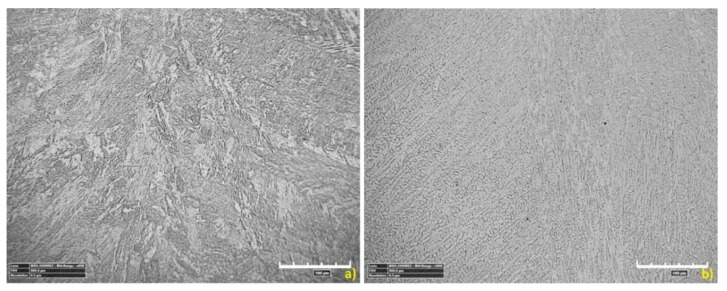
The weld structure approximately in the middle of the upper plate: (**a**) 1st configuration (S355J2 steel on top), (**b**) 2nd configuration (316L steel on top).

The weld composition is related to the mixture of the low-carbon and stainless steel alloying components, and the weld structure is a result of the solidification process of this newly formed material (Figure 17).

**Figure 18:**
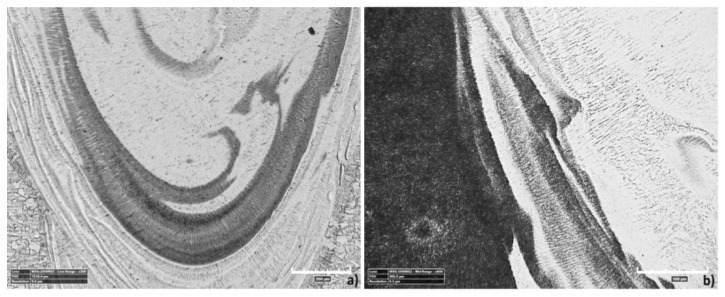
Dissimilarity in the weld structure, down plate: (**a**) 1st configuration (S355J2 steel on top), (**b**) 2nd configuration (316L steel on top).

The obtained structure has a dendritic build, however, in the case where low-carbon steel is the topsheet, the structure is composed of coarse-grained dendrites and acicular ferrite content. Meanwhile, for the configuration where stainless steel is the topsheet, the observed structure has a typical pillar dendritic build. In the 1st case, an austenitic-ferritic structure can be observed, however, in the 2nd case, there is only an austenitic structure. Moreover, fusion zones in the bottom sheet showed regions with non-uniform structures (Figure 18), according to region S1 (Figure 6) [66,67].

**Figure 19:**
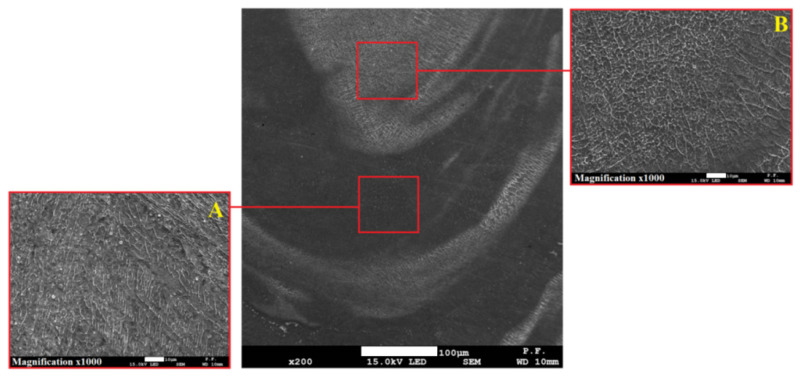
The weld structure approximately in the middle of the down plate, 2nd configuration (316L on top), with identified differences in structure (central picture) and the enlarged areas: (**A**)—pillar dendritic structure, (**B**)—cellular dendritic structure.

The SEM analysis showed differences in the weld structures, where separate areas inside the FZ were identified (Figure 19A,B). In area A, a pillar-dendritic structure was observed, while in area B, a dendritic cellular structure dominated.

**Figure 20:**
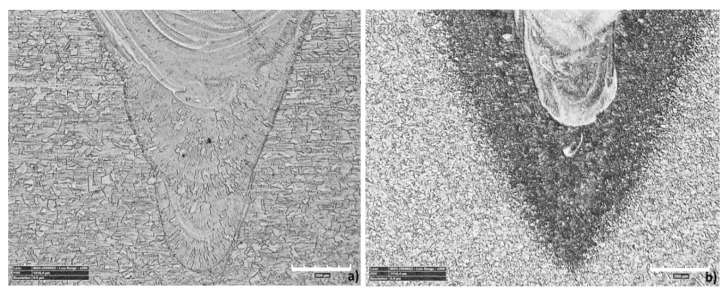
The fusion line of the weld in the down plate: (**a**) 1st configuration (S355J2 steel on top), (**b**) 2nd configuration (316L steel on top).

In the root of the weld (region S1), where the weld penetration was achieved, some differences were observed. The bottom weld area in austenitic steel had a structure similar to that of the BM (Figure 20a), while in ferritic-pearlitic steel, the structure was similar to that of the HAZ (Figure 20b).

**Figure 21:**
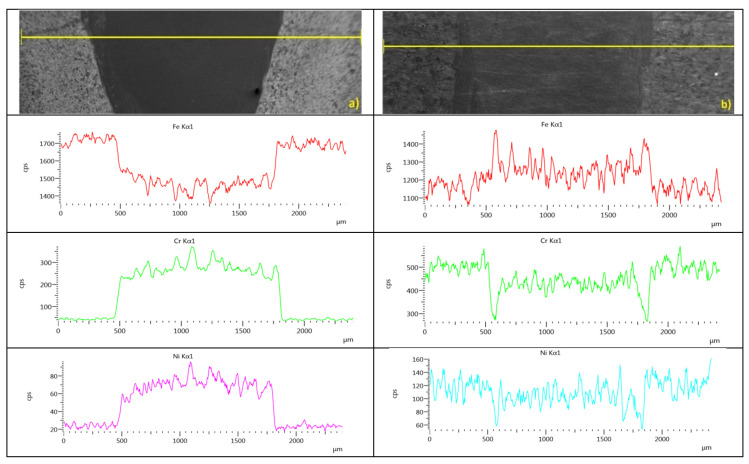
Iron, chromium, and nickel distribution according to S3 region: (**a**) 1st configuration, (**b**) 2nd configuration.

The non-uniform fusion zone structurewas confirmed through the EDS analysis. Therefore, according to the defined regions (presented in Figure 6), important differences in chemical compositionwere studied (Table 1) based on iron, nickel, and chromium distributions in the weld cross-section (Figure 21).

**Figure 22:**
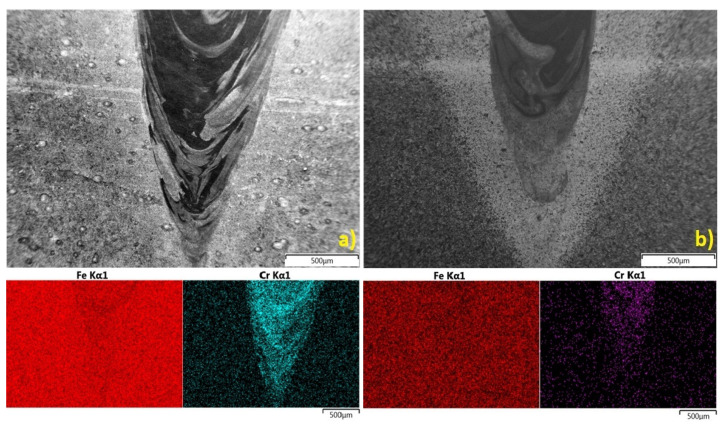
Bottom fusion zone iron and chromium map distribution (region S1): (**a**) 1st configuration (S355J2 steel on top), (**b**) 2nd configuration (316L steel on top).

The uniform distribution in the topsheet FZ proves the high mixture factor in this region, but the distribution in the bottomsheet FZ is more problematic (Figure 22).

The linear distribution of Fe, Cr, and Ni in the upper plate (region S3) showed greater differences for the 1st configuration (low-carbon steel as the topsheet), while in the 2nd configuration, the distribution was more uniform, however, an analysis showed largedifferences in the fusion line region. Moreover, according to the iron and chromium distributions, the joints made in the 1st configuration showed low iron content and a largeamount of chromium, while in the 2nd configuration, the iron distribution was almost uniform, with slightly increased chromium content.

## 4. Discussion

The welded materials have important differences in thermo-physical properties related to their chemical composition (Table 1). Both materials are characterized by good weldability, but due to the differencesdiscussed above, joining them by welding is problematic, especially when we consider a lap joint with partial penetration in the bottom plate, as described in this paper.

Global observation showed differences in the weld builds for both assumed joint configurations. The macroscopic analysis showed a potentially non-uniform structure in the fusion zones. Therefore, in order to investigate the quality level of the joints and to choose which configuration gives better results, numerical analysis and then metallographic studies were carried out.

Based on the developed numerical model, calibrated in the preliminary research, the welding parameters for the sealed lap joint were estimated. According to the established boundary conditions and the programmed simulation parameters, welding speed equal to 1 m/min with 6 kW of output power results in the assumed partial penetration. The performed simulations provided results where the differences in the width of the face of the weld were less than 14.5% compared to the trial joint for the 1st configuration and 1.62% for the 2nd configuration. The difference in the weld depth, calculated and obtained via experimental welding for the 1stconfiguration, was equal to 0.85% and for the 2nd configuration, to 2.1%. The width of the overlap region resulted in a 4.9% mismatch, comparing the simulation and the trial joint results for the 1st configuration, and 6.9% for the 2nd configuration. The numerical model obtained gave accurate results of the estimated weld bead geometry, however, the mismatch in the weld width for the 1st configuration, where low-carbon S355J2 steel was the topsheet, resulted in a 14.5% difference. The numerical model did not include the Marangoni effect and the surface tension, which has a great impact on the face of weld geometry. However, based on the small differences in other (Figure 7 and Figure 8) weld geometries, the established model gave realistic results and was used for joint stress–strain analysis [68,69,70].

In order to define the properties of the obtained joints based on the simulation results, the distribution of the effective plastic strain and the maximum principal stress was studied according to the determined lines (Figure 9). The M lines define points distributed parallel to the weld, near the fusion and heat-affected zones lines, and D lines are related to the weld penetration depth and are distributed perpendicular to the weld axis. According to the M1 and M2 lines, the effective plastic strain analysis showed amaximum value equal to 0.035 and was similar for both joint configurations, however, in the 2nd case, the values increased more rapidly. For the 1st case, the increase was slower, however, after the end of the welding cycle (1.2 s), it was still rising in the cooling stage, to 2.6 s. For the M2 lines (located 3.6 mm from the weld axis), the effective plastic strains were much smaller and the maximum calculated value (which occurred in the 2nd configuration) did not exceed 0.015, while for the 1st configuration, it was equal to 0.03. The differences in the effective plastic strain can be related to the different values of thermal conductivity (curves from 0 to 1.2 s), which for low-carbon steel resulted in a higher accumulation of thermal energy in the fusion zone and phase transformation (after 1.2 s) in the cooling stage [71].

The maximum principal stress values across the Dlines showed that the stress characteristic was divided into two separate cycles. The first was related to heating and cooling during movement of the HS, however, this value did not exceed 150 MPa. The second was related to material cooling, when the heat source was turned off. The maximum principal stress increased on the surface of the topsheet and its value ranged from 60 to 290 MPa for the 1st configuration and 20 to 320 MPa for the 2nd (the curve characteristics were similar for all measurement points). In the center line (D2), a greater stress value occurred in joint configuration number 1 and was equal to 540 MPa, while for the 2nd configuration, the value did not exceed 400 MPa. For the D3 lines, the values were less than 240 MPa (1st configuration) and equal to 200 MPa (2nd configuration). The maximum principal stress had a greater value in the measurement points located closer to the weld axis, withthegreatest difference occurring across the D2 line, where at points 5 and 6 they were more than 5.5 times greater than in points 7 and 8. The calculated maximum principal stress had a greater value in the 1st configuration and was related to the thermal gradient and the phase transformation of low-carbon steel [72,73].

A metallographic analysis confirmed the typical structure of the welded materials in the BM region, austenitic in 316L steel and ferritic-pearlitic in S355J2 steel. Significant differences in the heat-affected zones were observed. In stainless steel, the HAZ was very narrow and no growth of austenite grains was observed, while the elongated grains of the ferrite formed a discontinuous network around the austenite grains. These regions occur in steels with the structure of a native material consisting of austenite with the participation of ferrite δ. At high temperatures near the fusion line, the transformation of γ→δ occurs, which begins in the existing ferrite δ grains and progresses in areas with increased chromium concentration. After re-cooling, these areas do not achieve an equilibrium structure and the proportion of ferrite δ is increased. The HAZ of low-carbon steel is considerably wider and is made up of threeregions: an overheated zone, a normalization zone, and a partial recrystallization zone. These phenomena are related to phase transformation and structural changes during the solidification and cooling process. The obtained HAZs (including microstructure and geometry) of welded materials are typical for this steel grade welded using the LBW method [74].

In the global observation, some non-uniform microstructure of the fusion zones was detected, therefore, an extended weld microstructure analysis was carried out. The structure observed in the topsheet FZ had a typical uniform dendritic build, however, in the 1st configuration, coarse-graineddendritescontaining acicular ferrite were observed, while in the 2nd configuration, a typical pillar-dendritic build occurred. When the keyhole only partially penetrates the bottom workpiece, the influence of the flow field is clearly evident from the corresponding solidified structure. In the case of partial penetration, discrete growth bands occurred in the entire solidified weld bead, suggesting severe fluctuations in the flow field and the growth process [75]. On the contrary, in the topsheet, a full penetration keyhole was performed, where a columnar structure and an equiaxed zone along the centerline, similar to that shown in Figure 17b, can be observed. Discrete growth bands or striations occurred occasionally in this case, but the structural pattern was maintained within these bands. It should be noted that the laser output power in both cases was similar.

The fusion zone structure in the overlap region are similar to those observed in the FZ upper region, however, in the bottom plate, non-uniform structures were identified (Figure 18). There were differences in the fusion structure (Figure 19), where a pillar-dendritic build in region A and a cellular-dendritic structure in region B were observed. In the root of the weld, at the bottom of the fusion zone where weld penetration reaches, the structure was similar to the BM for stainless steel and the HAZ structure for low-carbon steel. In this region, the temperature exceeded the melting point, but only slightly, and according to the direction of heat diffusion in the XYZ axis, the heat flow to the BM was higher [76].

Homogenous weld builds are related to the uniform distribution of chemical elements. The welded materials have important differences in chemical composition, where S355J2 steel has a high content of iron and only trace amounts of chromium and nickel, while stainless steel contains more than 16% chromium, 11% nickel, and a balanced content of iron. Therefore, an analysis of defined regions in the cross-section of the welds was performed based on the linear and map distribution of these elements. The study showed bigger differences between the BM and the FZ in the distribution of Fe, Cr, and Ni in the 1st configuration. A clear boundary between the base material and the fusion zone was observed, however, the distribution curve had an almost vertical characteristic. For the 2nd configuration, the differences were smaller, with peak changes along the fusion line, which is related to chromium diffusion and concentration across the fusion line (phase transformation and increasing content of ferrite δ). An EDS analysis of the FZ bottom region showed further differences, particularly in chromium and iron distribution [77]. For the 1st configuration, small amounts of iron were detected and considerably greater differences in the detection of chromium were observed (Figure 22a). In the 2nd joint configuration, the iron distribution in the fusion zone and the base material was almost uniform, and some differences in chromium detection were observed (Figure 22b).

## 5. Conclusions

The study of laser lap joint welding, where low-carbon S355J2 and stainless 316L steel were joined together in two configurations (1st with low-carbon steel on the top, 2nd with stainless steel on the top), showed significant differences in the obtained results. According to the developed numerical model and the established boundary conditions, an accurate match of the simulated and experimental results of weld geometry was obtained. Assumingthat the obtained model gave accurate results, a numerical stress–strain analysis was performed. Higher values of effective plastic strain occurred in the 2nd configuration, however, the maximum principal stress values were greater in the 1st configuration. Greater differences in the measurement points occurredfor the configuration with low-carbon steel placed on the top. The microstructure analysis showed further differences, most of all in the fusion zone structure, where in the bottom plate, two separate structures were detected. A more uniform structure was observed in the 1st configuration, however, greater differences in Cr, Ni, and Fe distribution between the FZ and the BM were detected in this configuration. No porosity, cracks, or welding defects were identified, therefore, according to therequirement of PN-EN ISO 13919-1, a B quality level was assumed. According to the performed study, and based on the maximum principal stress distribution, differences in the weld structure, and the distribution of the alloying elements, the second configuration showed better properties. Therefore, the second configuration, where stainless steel is placed on the top, was chosen as a dominant joint. The microstructure study showeda non-uniform mixture of welded material in the root of the weld, however, no welding defects or precipitations in the inter-plate region were observed. This study confirmsthe impact of the welded material configuration on joint properties and shows which configuration ensures the lowest stress and strain concentration and a more uniform weld structure. Therefore, according to:

− calculated maximum principal stress value.
− lower differences in the chemical element distribution in theweld.
− more uniform structure and fusion rate in the weld.

When laser lap welding of S355J2 and 316L steels is considered, the configurationin which stainless steel is placed on top provides better joint properties and is recommended.The presented results showed that when ahigh-qualitysteel joint with good strength characteristics is required, such as pipeline components or crude oil storage tanks, the welding position should be related to the stainless steel side.

Further investigation of this joint type is required, therefore, the possibility of usingtwin spot laser welding optics is planned. Theresearch will also be extended to mechanical tests, including the tensile strength and hardness tests.

## Figures and Tables

**Table 1:** Chemical composition of materials used.

Material	Elements (wt %)										
	C	Mn	Si	P	S	Cr	Ni	Al	Fe	Cu	CEV
**S355J2**	0.17	1.6	0.02	0.017	0.011	0.02	-	0.05	98.1	0.06	0.45%
**316L**	0.018	1.57	0.48	0.04	0.002	16.7	11.2	-	balance	-	-
